# Supplementary material for: Objective Assessment of Sleep Patterns among Night-Shift Workers: A Scoping Review
Source: Int J Environ Res Public Health. 2021 Dec 15;18(24):13236. doi: 10.3390/ijerph182413236 (PMC8701940; doi:10.3390/ijerph182413236)
Supplement: Supplementary file 1 [file ijerph-18-13236-s001.zip › ijerph-1384223-supplementary.pdf]

Search expressions

CINAHL

S28 S27 AND LA eng 227

S27 S26 AND DT 19910101-20201231 247

S26 S6 AND S13 AND S25

S25 S14 OR S15 OR S16 OR S17 OR S18 OR S19 OR S20 OR S21 OR S22 OR S23 OR S24 1098454

S24 TI wearable device OR AB wearable device OR SU wearable device 871

S23 TI smart OR AB smart OR SU smart 7832

S22 TI mobile OR AB mobile OR SU mobile 28156

S21 TI actigraph OR AB actigraph OR SU actigraph 1594

S20 TI actiware OR AB actiware OR SU actiware 3

S19 TI sensor OR AB sensor OR SU sensor 9380

S18 TI measurement OR AB measurement OR SU measurement 263532

S17 TI assessment OR AB assessment OR SU assessment 706902

S16 TI device OR AB device OR SU device 90904

S15 TI tool OR AB tool OR SU tool 169332

S14 TI band OR AB band OR SU band 11528

S13 S7 OR S8 OR S9 OR S10 OR S11 OR S12 79147

S12 TI sleep disorder OR AB sleep disorder OR SU sleep disorder 6707

S11 TI sleep problem OR AB sleep problem OR SU sleep problem 4036

S10 TI sleep deprivation OR AB sleep deprivation OR SU sleep deprivation 4704

S9 TI sleep disturbance* OR AB sleep disturbance* OR SU sleep disturbance* 7279

S8 TI insomnia OR AB insomnia OR SU insomnia 11903

S7 TI sleep OR AB sleep OR SU sleep 73345

S6 S1 OR S2 OR S3 OR S4 OR S5 6306

S5 TI rotating work OR AB rotating work OR SU rotating work 155

S4 TI nightwork OR AB nightwork OR SU nightwork 7

S3 TI night work OR AB night work OR SU night work 791

S2 TI shiftwork OR AB shiftwork OR SU shiftwork 4149

S1 TI shift work OR AB shift work OR SU shift work 2871

Cochrane Library

#1 (shift work):ti,ab,kw 996

#2 (shiftwork):ti,ab,kw 83

#3 (night work):ti,ab,kw 935

#4 (nightwork):ti,ab,kw 11

#5 (rotating work):ti,ab,kw 99

#6 #1 OR #2 OR #3 OR #4 OR #5 1662

#7 MeSH descriptor: [Sleep] explode all trees 5709

#8 (sleep):ti,ab,kw 37591

#9 sleep disturbance* 5314

#10 sleep problem 2223

#11 sleep disorder 8638

#12 insomnia 11584

#13 MeSH descriptor: [Sleep Deprivation] explode all trees 754

#14 (sleep deprivation):ti,ab,kw 1716

#15 #7 OR #8 OR #9 OR #10 OR #11 OR #12 OR #13 OR #14 44586

#16 (band):ti,ab,kw 4760

#17 (device):ti,ab,kw 44131

#18 (assessment):ti,ab,kw 229872

#19 (measurement):ti,ab,kw 91223

#20 (sensor):ti,ab,kw 3354

#21 (actiware):ti,ab,kw 1

#22 (actigraph):ti,ab,kw 946

#23 (mobile):ti,ab,kw 9455

#24 (smart):ti,ab,kw 3160

#25 MeSH descriptor: [Wearable Electronic Devices] explode all trees 435

#26 (wearable device):ti,ab,kw 481

#27 (tool):ti,ab,kw 24730

#28 #16 OR #17 OR #18 OR #19 OR #20 OR #21 OR #22 OR #23 OR #24 OR #25 OR #26 OR #27 358951

#29 #6 AND #15 AND #28 with Cochrane Library publication date Between Jan 1991 and Dec 2020 221

Pubmed

((((((shift work[All Fields]) OR (shiftwork[All Fields])) OR (night work[All Fields])) OR (nightwork[All Fields])) OR (work[MeSH Terms])) OR (rotating work[All Fields])) Filters: Publication date from 1991/01 to 2020/12

AND

((((((sleep[MeSH Terms]) OR (insomnia[All Fields])) OR (sleep disturbance*[All Fields])) OR (sleep deprivation[MeSH Terms])) OR (sleep problem[All Fields])) OR (sleep disorder[All Fields])) OR (sleep quality)[All Fields]) Filters: Publication date from 1991/01 to 2020/12

AND

((((((((((band[All Fields]) OR (tool[All Fields])) OR (device[All Fields])) OR (assessment[All Fields])) OR (measurement)) OR (sensor[All Fields])) OR (actiware[All Fields])) OR (actigraph [All Fields])) OR (mobile[All Fields])) OR (smart[All Fields])) OR (wearable device[All Fields])) Filters: Publication date from 1991/01 to 2020/12

Scopus

28 limit 27 to pub year 1991–2020 878

27 limit 26 to english language 972

26 #6 and #13 and 25 1033

25 #14 or #15 or #16 or #17 or #18 or #19 or #20 or #21 or #22 or #23 or #24 15181681

24 TITLE-ABS-KEY (“wearable device”)

23 TITLE-ABS-KEY (“smart”) 346945

22 TITLE-ABS-KEY (“mobile”) 810723

21 TITLE-ABS-KEY (“actigraph”) 3758

20 TITLE-ABS-KEY (“actiware”) 49

19 TITLE-ABS-KEY (“sensor”) 1282163

18 TITLE-ABS-KEY (“measurement”) 5038678

17 TITLE-ABS-KEY (“assessment”) 3950616

16 TITLE-ABS-KEY (“device”) 2976792

15 TITLE-ABS-KEY (“tool”) 2514859

14 TITLE-ABS-KEY (“band”) 1374244

13 #7 or #8 or #9 or #10 or #11 or #12 351235

12 TITLE-ABS-KEY (“sleep disorder”) 75747

11 TITLE-ABS-KEY (“sleep problem”) 8137

10 TITLE-ABS-KEY (“sleep deprivation”) 19279

9 TITLE-ABS-KEY (“sleep disturbance*”) 21173

8 TITLE-ABS-KEY (“insomnia”) 70567

7 TITLE-ABS-KEY (“sleep”) 311044

6 #1 or #2 or #3 or #4 or #5 9388

5 TITLE-ABS-KEY (“rotating work”) 78

4 TITLE-ABS-KEY (“nightwork”) 86

3 TITLE-ABS-KEY (“night work”) 2398

2 TITLE-ABS-KEY (“shiftwork”) 1205

1 TITLE-ABS-KEY (“shift work”) 7196

Web of Science

#28 limit 27 to pub year 1991–2020 1140

#27 limit 26 to English OR Korean language 1278

#26 #6 AND #13 AND #25 1323

#25 #14 OR #15 OR #16 OR #17 OR #18 OR #19 OR #20 OR #21 OR #22 OR #23 OR #24 6875057

#24 TS=wearable device 15219

#23 TS=smart 112635

#22 TS=mobile 269759

#21 TS=actigraph 3057

#20 TS=actiware 11

#19 TS=sensor 556319

#18 TS=measurement 2391194

#17 TS=assessment 1556733

#16 TS=device 1045298

#15 TS=tool 1318255

#14 TS=band” 815355

#13 #7 OR #8 OR #9 OR #10 OR #11 OR #12 235335

#12 TS=sleep disorder 59278

#11 TS=sleep problem 20395

#10 TS=sleep deprivation 14471

#9 TS=sleep disturbance* 0

#8 TS=insomnia 28665

#7 TS=sleep 224493

#6 #1 OR #2 OR #3 OR #4 OR #5 116384

#5 TS=rotating work 17888

#4 TS=nightwork 51

#3 TS=night work 11928

#2 TS=shiftwork 787

#1 TS=shift work 92350
